# Radicular pain following low lumbar basivertebral nerve ablation

**DOI:** 10.1016/j.inpm.2023.100270

**Published:** 2023-08-03

**Authors:** Jacob Benrud, Yakov Vorobeychik

**Affiliations:** Department of Anesthesiology and Perioperative Medicine, Penn State Health. Milton S. Hershey Medical Center, Hershey, PA, 17036, USA

Dear Editor:

Basivertebral Nerve Ablation (BVNA) has emerged as a promising procedure for the treatment of lumbosacral vertebrogenic pain [1,2], which is prevalent among young and middle-aged patients (average age 47 years) [2]. Most commonly, this procedure is performed at the L5 and S1 levels [[3], [4], [5]].

Leg pain, paresthesia, or motor deficits following BVNA have been reported from 1.6% to 11% [4,[6], [7], [8]]. In the Koreckij et al. study, 14 of 127 patients (11%) developed post-ablation radicular leg pain that lasted 48.5 days on average and mainly occurred following procedures performed at the L5/S1 level. An independent review of post-ablation MRI concluded that except for one undetermined case, all radicular pain events occurred due to pedicle breach [6]. There were no observed effects related to learning curve patterns, anesthesia type, proceduralist specialty, or experience [6]. Leg pain and paresthesia were also observed in 6 of 51 (10.7%) study participants in the Khalil et al. study, which resolved in all but two (3.9%) participants by three months post-intervention [4]. Trumees et al. also reported leg pain in two of 28 (7.1%) patients [7]. Fischgrund et al. in their original study, noted that 4 of 255 (1.6%) of BVNA procedures resulted in transient motor or sensory deficits [9].

Practitioners usually use the transpedicular approach under fluoroscopic guidance to target the basivertebral nerve. It is steered from the superior-lateral pedicle at the entry to the inferior-medical pedicle at the exit from the pedicle into the vertebral body. To accurately reach the target, it is suggested to deploy curved nitinol stylet through the RF introducer trocar and direct it ventromedially to the target after the trocar passes through the pedicle [9]. The anatomy of lumbar vertebral bodies changes from concave (kidney shape) at the upper lumbar levels to oval at the L5 level [1]. In the lateral view, the oval shape of L5 may create the illusion that the needle tip is already past the pedicle, whereas it is still inside it. As a consequence, the operator may turn the RF cannula medially prematurely, breaking through the infero-medial wall of the pedicle into the lateral recess and damaging the traversing L5 nerve (Fig. 1).

**Fig. 1:**
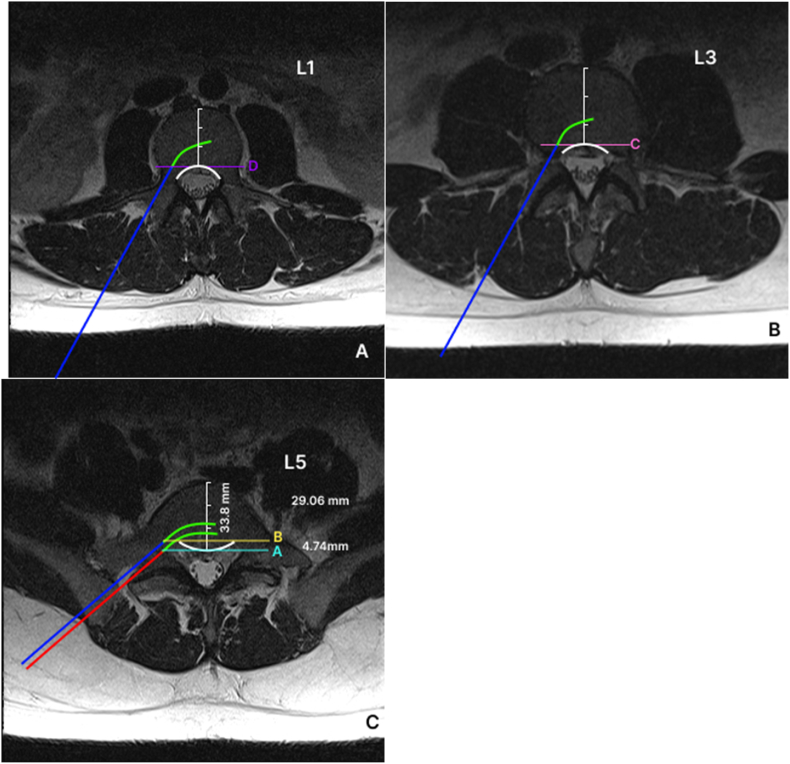
(A, B) Axial cut of L1 (A) and L3 (B) on MRI illustrating the concave or kidney shape of the vertebral body (white line). Blue line represents a topical path of the introducer through the pedicle. Green curved line represents the approximate path of the curved stylet that is usually introduced just as the posterior wall of the vertebral body is reached on a lateral fluoroscopic image. Lines C and D represent the posterior wall of the vertebral body as would be seen in a lateral fluoroscopic image. BVNA is approved for L3 to S1 vertebral levels. We included an L1 image to show a gradual transition of the lumbar vertebral body shape from concave at the upper lumbar levels to oval at the L5 level. Furthermore, the current approval may be expanded at a future date to include vertebral levels cephalad from L3. (C) Axial cut of L5 on MRI illustrating the convex shape of the L5 vertebral body (white line). Red line represents an approximal path of an introducer with curved green line representing the path of curved stylet. Note that in this illustration the curved stylet comes dangerously close to the L5 spinal nerve root and potentially can breach the pedicle. The blue line represents a safer path for an introducer accounting for the convex shape of the L5 vertebral body. Line A represents the posterior wall of the vertebral body whereas line B represents the anterior border of the pedicle. Introduction of the curved stylet at this more ventral location prevents breaching the pedicle and damaging the passing nerve. (For interpretation of the references to colour in this figure legend, the reader is referred to the Web version of this article.)

By reviewing advanced imaging and calculating the relative distance from the posterior wall of the vertebral body to the anterior border of the pedicle, percentage distance can be calculated for an individual patient, which then can be extrapolated for use under fluoroscopy (Fig. 2). The L5 vertebral body can be examined with an MRI or CT scan in axial view. We suggest the following formula for this calculation:

$$Safe\;Entry\;Point\;Relative\;Distance=\frac{AP\;Distance\;of\;Vertebral\;Body-AP\;Distance\;of\;Pedicle\;Entry\;Point}{AP\;Distance\;of\;Vertebral\;Body}\;x\;100\%$$

**Fig. 2:**
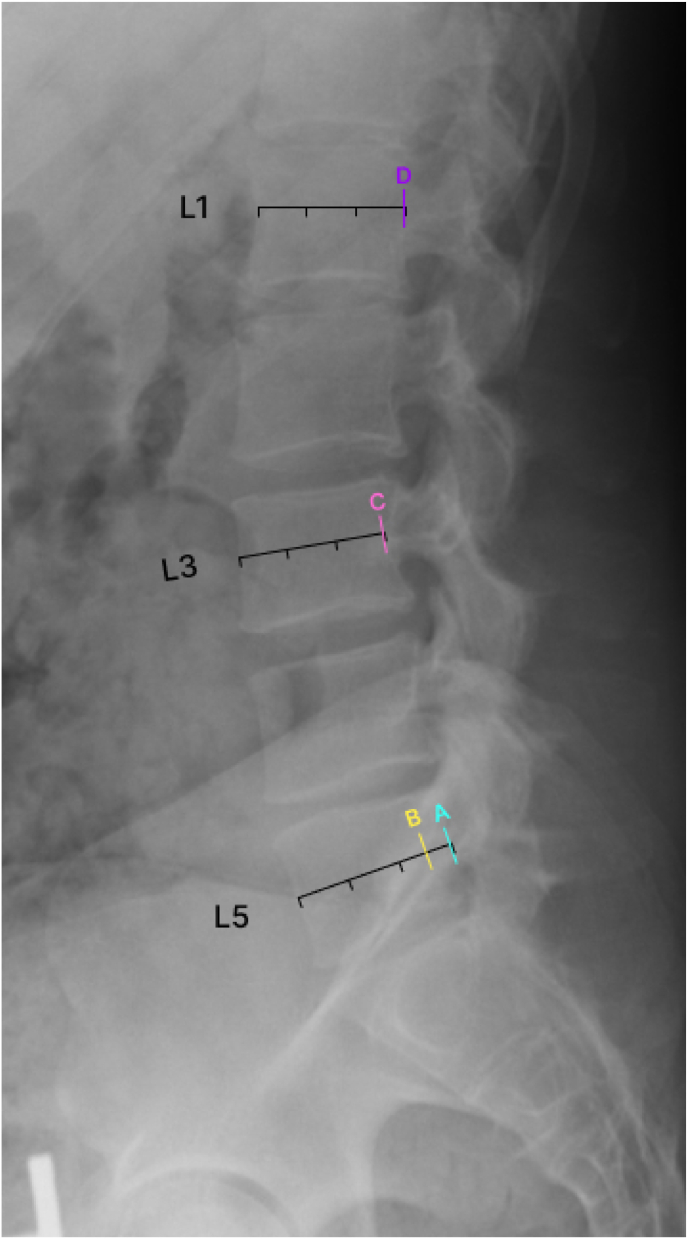
The lateral view radiograph illustrates where lines A, B, C, and D would appear on this image. Note that line A seems to be a safe location to begin introducing the curved stylet; however, if a curved stylet is introduced here, it will likely encounter, or be very close to, the spinal nerve root. Line B is more ventral and should be the point at which the curved stylet is introduced. The discrepancy between lines A and B is due to the convex shape of the L5 vertebral body. Lines C and D represent the posterior wall of the L1 and L3 vertebral bodies respectively.

The first point of measurement should be the AP distance of the vertebral body at the midpoint. The second point of measurement should be the AP distance at the midpoint of the line connecting both pedicles where they meet the vertebral body (AP distance of pedicle entry point in the above formula). The difference in these distances divided by the AP distance of the vertebral body will yield a ratio to be multiplied by 100% to give the relative distance from the posterior vertebral border, which needs to be accounted for when viewing the lateral fluoroscopic image. This measure should allow calculating the distance from the apparent vertebral body posterior border on a lateral fluoroscopic image to the ventral border of the pedicle, wherein it becomes safe to introduce the curved stylet without fear of breaching the pedicle and causing nerve damage. As an example, we present the MRI images of a typical patient. For the presented L5 image, the calculations using the formula are as follows:

$$Safe\;Entry\;Point\;Relative\;Distance=\frac{33.8mm-29.06\;mm}{33.8mm}\;x\;100\%=14.0\%$$

Therefore, there should be a ventral correction of 14% of the AP distance on a lateral X-ray image before a curved stylet should be introduced.

We strongly recommend reviewing available advanced imaging studies before performing BVNA at any level. The unique anatomy of L5 requires special attention and pre-operative planning to reduce the risk of pedicle breach and nerve root injury. We recognize that our suggestion will need to be studied in more detail before it can be adopted in pain management practice; we provide a theory of probable causation of radicular pain that has been described in multiple studies and the safer way of performing this procedure to avoid this complication.

We would like to show our gratitude to Mr. Matthew J. Schilling for his help with designing the electronic artwork for this manuscript.

## Declaration of competing interest

The authors declare that they have no known competing financial interests or personal relationships that could have appeared to influence the work reported in this paper.
